# COVID-19 and the ageing workforce: global perspectives on needs and solutions across 15 countries

**DOI:** 10.1186/s12939-021-01552-w

**Published:** 2021-10-07

**Authors:** Sabrina Pit, Malcolm Fisk, Winona Freihaut, Fashola Akintunde, Bamidele Aloko, Britta Berge, Anne Burmeister, Adriana Ciacâru, Jürgen Deller, Rae Dulmage, Tae Hwa Han, Qiang Hao, Peter Honeyman, Peter C. Huber, Thomas Linner, Stefan Lundberg, Mofoluwaso Nwamara, Kamolpun Punpuing, Jennifer Schramm, Hajime Yamada, Jason C. H. Yap

**Affiliations:** 1 University Centre for Rural Health, School of Medicine, Western Sydney University, Lismore, NSW 2480 Australia; 2 Faculty of Medicine and Health, University of Sydney, Lismore, NSW 2480 Australia; 3 NSW Rural Doctors Network, Newcastle, NSW 2303 Australia; 4 De Montfort University, Gateway House, The Gateway, Leicester, LE1 9BH UK; 5 University of Melbourne, Melbourne, VIC Australia; 6 Society for Human Resources (SHRM), Chartered Institute of Personnel Management of Nigeria, Lagos, Nigeria; 7 AARP Public Policy Institute, AARP Public Policy Institute, 601 E St, NW, Washington, DC, 20049 USA; 8 Department of Organisation and Personnel Management, Rotterdam School of Management (RSM), Erasmus University Rotterdam, Mandeville Building T10-50, Burgemeester Oudlaan 50, 3062 PA Rotterdam, The Netherlands; 9 Trade Union in the Field of Social Assistance & Child Protection, Federation COLUMNA, Vespasian Street, 39, București, Romania; 10 Institute of Management & Organization, Leuphana University, Lüneburg, Germany; 11 Ontario, Canada; 12 College of Medicine, Health IT Center, Yonsei University Health System, Yonsei University, 50-1 Yonsei-ro, Seodaemun-gu, Seoul, 120-752 South Korea; 13 Beijing Century Myway Education Technology Inc., 209 Tower A, Heqiao Building, Guanghua Road, Chaoyang Dist, Beijing, China; 14 School of Public health, Hebrew University, Hadassah Ein Kerem, Jerusalem, Israel; 15 Staff Unit Organization, Standardization, Research Communication, Austrian Association Supporting the Blind and Visually Impaired, Schlosshofer Straße 2-6, 1210 Wien, Austria; 16 Technical University Munich (TUM), Arcisstraße 21, 80333 Munich, Germany; 17 The Swedish Consumers’ Association, SCA member of ANEC the European Consumer Voice in Standardisation, Vantörsvägen, 209 125 55 Älvsjö, Sweden; 18 Society for Human Resources (SHRM) Nigeria, Lagos, Nigeria; 19 Research Strategic Planning and Management Division, National Science and Technology Development Agency, 11 Thailand Science Park, Phahonyothin Road, Khlong Nueng, Khlong Luang, Pathum Thani, 12120 Thailand; 20 Financial Security, AARP Public Policy Institute, AARP Public Policy Institute, 601 E St, NW, Washington, DC, 20049 USA; 21 Tokyo University, 5-28-20 Hakusan, Bunkyo, Tokyo, 112-0001 Japan; 22 Saw Swee Hock School of Public Health, National University of Singapore, 12 Science Drive 2, #10-00 Tahir Foundation Building, Singapore, 117549 Singapore

**Keywords:** Ageing, COVID-19, health equity, employment, planning, workforce, international, solution

## Abstract

**Background:**

COVID-19 has a direct impact on the employment of older people. This adds to the challenge of ageism. The World Health Organization has started a worldwide campaign to combat ageism and has called for more research and evidence-based strategies that have the potential to be scaled up. This study specifically aims to identify solutions to combat the adverse effects of COVID-19 on the global ageing workforce.

**Methods:**

We present 15 case studies from different countries and report on what those countries are doing or not doing to address the impact of COVID-19 on ageing workers.

**Results:**

We provide examples of how COVID-19 influences older people’s ability to work and stay healthy, and offer case studies of what governments, organizations or individuals can do to help ensure older people can obtain, maintain and, potentially, expand their current work. Case studies come from Australia, Austria, Canada, China, Germany, Israel, Japan, Nigeria, Romania, Singapore, Sweden, South Korea, Thailand, United Kingdom (UK), and the United States (US). Across the countries, the impact of COVID-19 on older workers is shown as widening inequalities. A particular challenge has arisen because of a large proportion of older people, often with limited education and working in the informal sector within rural areas, e.g. in Nigeria, Thailand and China. Remedies to the particular disadvantage experienced by older workers in the context of COVID are presented. These range from funding support to encouraging business continuity, innovative product and service developments, community action, new business models and localized, national and international actions. The case studies can be seen as frequently fitting within strategies that have been proven to work in reducing ageism within the workplace. They include policy and laws that have increased benefits to workers during lockdowns (most countries); educational activities such as coaching seniorpreneurship (e,g, Australia); intergenerational contact interventions such as younger Thai people who moved back to rural areas and sharing their digital knowledge with older people and where older people reciprocate by teaching the younger people farming knowledge.

**Conclusion:**

Global sharing of this knowledge among international, national and local governments and organizations, businesses, policy makers and health and human resources experts will further understanding of the issues that are faced by older workers. This will facilitate the replication or scalability of solutions as called for in the WHO call to combat ageism in 2021. We suggest that policy makers, business owners, researchers and international organisations build on the case studies by investing in evidence-based strategies to create inclusive workplaces. Such action will thus help to challenge ageism, reduce inequity, improve business continuity and add to the quality of life of older workers.

## Background

Older people are one of the highest-risk groups for COVID-19, although the extent of risk may vary by country, region or population depending on factors such as the nature and scope of existing health care systems and prevalence of chronic conditions [1]. Being at a higher level of risk has a direct impact on older people’s employment. The global economic downturn, social isolation requirements and the health risk itself all play a part and increase the risk of health inequities for older people being worsened.

Ageing workforce can be defined as “the increase in the number of older people in the workforce”. The definition of an older worker varies by country, industry, and other factors [2]. Ageing can, furthermore, be viewed through different lenses. It is a complex phenomenon that is not only based on chronological age but is also associated with career paths, type of organization, biological and functional ageing. Furthermore, different views on ageing partly explain why countries have different formal retirement ages or none at all. Hence the impact of COVID-19 on ageing workers varies. Factors include individual resources, geographic location, the size of employing organizations and their sectors, the extent of digital access, skill disability and cognitive impairments and caring responsibilities.

A US study demonstrated the early and potential future effects of COVID-19 on the employment of older workers. The authors found that COVID-19 has disproportionately affected older workers - with unemployment rates rising to 15.4% for people aged 65 years and older compared to 13.0% for those aged 25-44 [3]. They reported that older workers are *‘often hit hard by recessions, and often harder than younger workers’,* causing for many a loss of employment leading to an increase in both social security claims and poverty. Overall, older peoples’ working lives may be shortened due to being unable to re-engage in the world of work.

Countries with limited social security provision can experience extra challenges. An Indian study of mostly unskilled workers [4] examined the impact of the lockdown that was a consequence of COVID-19 and reported that 86% of workers were engaged in work that had stopped. In particular, the authors demonstrated that income loss was greater for older laborers. They speculated that this may have been due to the early removal of older workers immediately in the early period of the pandemic.

The World Health Organisation Global Report on Ageism is important by virtue of its release during the pandemic. This initiated a campaign to combat ageism [5]. It stated that “*Ageism refers to the stereotypes (how we think), prejudice (how we feel) and discrimination (how we act) directed towards people on the basis of their age. It can be institutional, interpersonal or self-directed*.” The report found that both older and younger workers are more disadvantaged in the workplace and ageism is more likely to occur in certain professions or industry sectors - such as high tech or hospitality. Ageism, it noted, damages both people and the broader economy through reducing people’s ability to reach their full potential, leading to financial insecurity and poverty. Three strategies to reduce ageism are set out: investment in evidence-based strategies to combat ageism, improvements in research on ageism; and the building of a movement to change the narrative on aging. Ageism is, the report pointed out, common in the workplace and, together with COVID-19 may further adversely impact older workers.

We illustrate responses to the effects of COVID-19 on the global ageing workforce from fifteen different countries. These point to the most pressing issues and what each country is doing or not doing to assist older workers who are threatened or impacted by the pandemic. The case studies offer a number of potential remedies. The co-authors of this paper are all involved with and have relevant expertise relating to ageing in their respective countries and may be positioned to guide their governments regarding appropriate measures both during the pandemic and in its aftermath.

Closely aligned with this is the work of the International Organization for Standardization (ISO) Technical Committee TC 314 on ‘Ageing Societies’ that is developing an international standard on Age Inclusive Workforces. This is due for publication in 2022 [2]. The standard is being developed by experts from 20 countries, including representatives of industry and commercial bodies, governments, trade unions, consumers, academics and researchers, service providers, and carers. The new standard will provide recommendations for the provision and/or maintenance of quality, meaningful work that empowers the workers of all ages to be productive and add value to the employing organizations. We call on people to provide more case studies and commentaries to ensure that the Technical Committee is positioned to share ideas that may be replicable and help address the needs of older workers both during and after the pandemic.

This study specifically aims to: 1) illustrate different responses to the effects of COVID-19 on the global ageing workforce; and 2) offer a number of potential solutions to combat the adverse effects of COVID-19 on it.

## Methods

We present case studies from different countries and report what they are doing or not doing to address the issues that have arisen. We deliberately let countries choose their analytical template and to describe a case study of their choice. This, it was considered, could lead to a wider spread of ideas that might offer more innovative solutions whereby the inequity encountered by older workers could be redressed. Ageing workforce experts from the ISO Age Inclusive Workforce working group were initially invited to take part. Further experts were added from Israel, Nigeria and South Korea through personal contacts and suggestions. Unsuccessful attempts were made to include South American countries.

First an overview is presented of COVID-19 statistics in each country. Secondly, the case study is provided for each country presaged by a brief background, statement of the problem and potential solutions. Thirdly, a comparative analysis is provided to identify commonalities, differences and note the range of solutions provided.

## Results

### Comparison and context

**Fig. 1:**
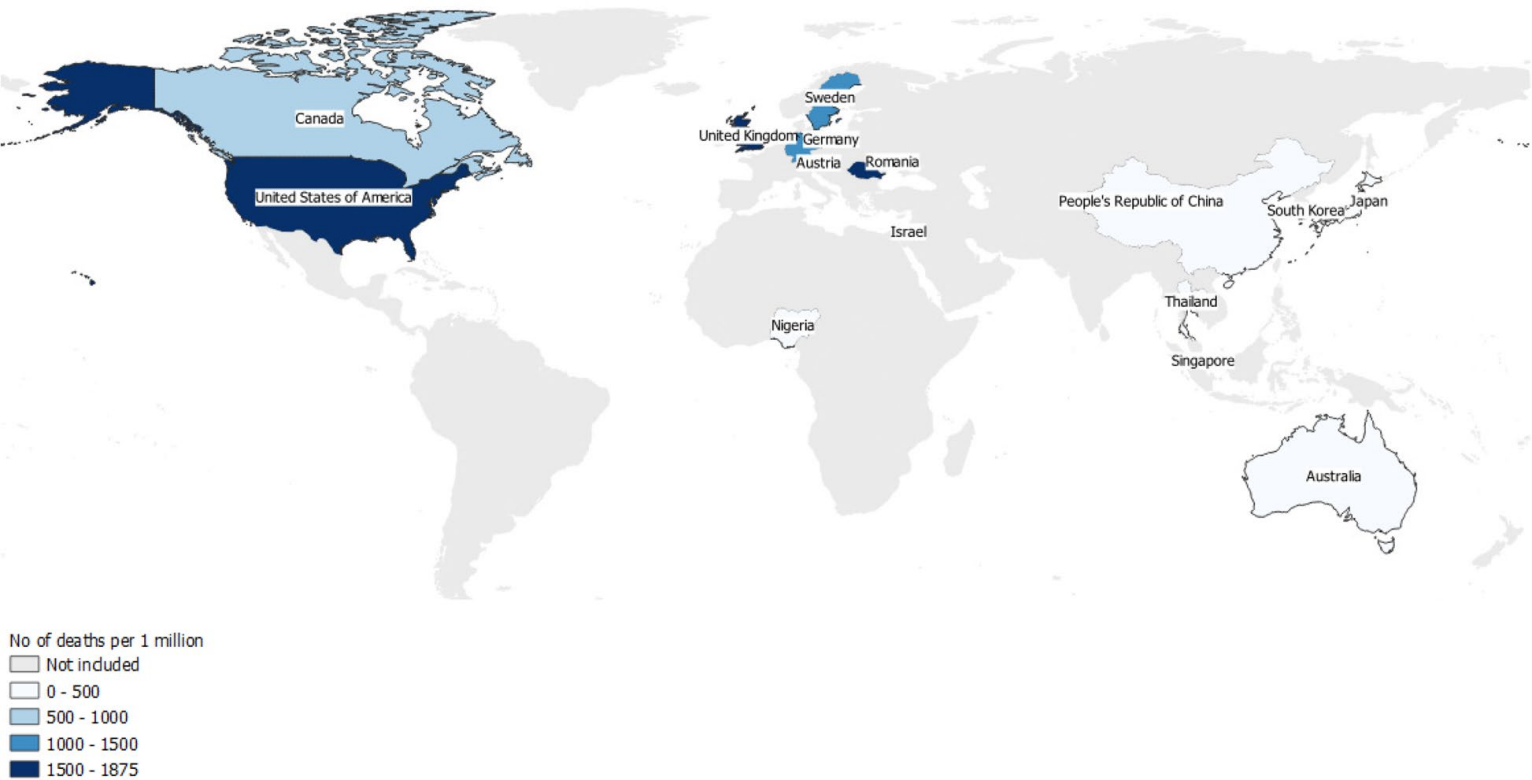
Number of deaths due to COVID-19 per 1 million population per 19 June 2021. Source: https://www.worldometers.info/coronavirus/#countries 19 June 2021

An overview of COVID-19 and ageing workforce indicators in each country provide context for the case studies. Fig. 1 provides an overview of the number of COVID-19 deaths per 1 million population for each country as at 19th June 2021. Countries with fewer than 500 deaths per 1 million population at this date included China, Singapore, Nigeria, Thailand, Australia, South Korea, and Japan. This was followed by Canada and Israel with both close to 700 deaths per 1 million population. The third group included Germany, Austria and Sweden with deaths per 1 million people ranging between 1000 and 1500, with the highest rates being in Romania, the USA and the UK.

Table 1 provides further details of cases, deaths and COVID-19 tests performed. All can be seen as experiencing active cases but there is wide variation in the numbers, with Australia, at 140, having the least number of active cases and the United States over 5 million. Nigeria performed the least testing with about 10,000 COVID-19 tests per 1 million population, whilst Austria has performed almost 5.5 million tests per 1 million Australians.

**Table 1:** Statistics of cases, deaths and COVID-19 tests as at 19 June 2021^a^

Country	Total cases	Total deaths	Active cases	Total cases per 1 million	Total deaths per 1 million	Total tests per 1 million	Total population
Australia	30,322	910	140	1176	35	761,932	25,784,225
Austria	649,309	10,677	3088	71,700	1179	5,541,560	9,055,861
Canada	1,407,269	26,023	12,797	36,975	684	947,249	38,060,421
China	91,564	4636	503	64	3	111,163	1,439,323,776
Germany	3,728,559	90,877	39,582	44,365	1081	750,711	84,041,960
Israel	839,769	6428	280	90,046	689	1,582,470	9,326,000
Japan	780,898	14,320	23,730	6193	114	124,488	126,103,046
Nigeria	167,155	2117	1498	792	10	10,574	211,030,850
Romania	1,080,140	32,212	3009	56,511	1685	498,312	19,113,828
South Korea	150,720	1997	6338	2937	39	201,185	51,311,703
Singapore	62,382	34	361	10,583	6	2,173,030	5,894,604
Sweden	1,084,636	14,537	29,521	106,757	1431	1,025,907	10,159,870
Thailand	214,449	1609	31,482	3065	23	116,190	69,968,690
United Kingdom	4,610,893	127,956	185,180	67,579	1875	2,931,656	68,229,291
United States	34,393,269	616,920	5,100,420	103,323	1853	1,496,767	332,870,823

^a^Source: https://www.worldometers.info/coronavirus/#countries 19 June 2021

Table 2 shows that the average life expectancy at birth is above 80 years for most of the fifteen countries, with only the US (79), Thailand (78), China (77), Romania (77) and Nigeria (56) below this level.

**Table 2:** Retirement age and employment/ population ratio for people aged 50 years and over

Country	Life expectancy (years)^1^	Share of employment in 2019 of total working population in % by age (years)^2^					Retirement age in 2018^3^	
		50-54	55-59	60-64	65-69	70-74	Men	Women
Australia	84	9.6	8.6	6.1	2.7 ^**2**^	–	65	65
Austria	82	13.9	11.3	3.9	0.9	0.5	65	60
Canada	83	10.6	10.1	6.9	3.0	1.2	65	65
China	77	–	–	–	–	–	60	55
Germany	82	13.9	12.9	8.1	2.0	0.7	65.5	65.5
Israel	83	8.8	7.8	6.2	3.8	1.5	67	62
Japan	85	10.9	9.4	7.9	6.4	4.1	65	64
Nigeria	56	–	–	–	–	–	60	60
Romania	77	12.5	8.4	5.0	2.0	1.1	65	61
South Korea	84	12.3	11.5	8.0	4.4	2.5	61	61
Singapore	84	–	–	–	–	–	62	62
Sweden	83	11.8	10.1	7.8	2.6	1.2	65	65
Thailand	78	–	–	–	–	–	60	60
United Kingdom	82	11.8	10.0	6.4	2.4	1.0	65	62.7
United States	79	10.0	9.7	7.3	3.7	1.7	66	66

^1^Source: Data extracted on 20 Jun 2021 from https://www.worldometers.info/demographics/life-expectancy/#countries-ranked-by-life-expectency

^2^Source: Data extracted on 20 Jun 2021from OECD.Stat. https://stats.oecd.org/Index.aspx?QueryId=64196# The table shows the age composition (as a percentage of all ages) of the population that is in employment. ^2^ Latest data available was 2019. Not available: China, Nigeria, Singapore and Thailand

^3^Source: OECD estimates derived from the European and national labour force surveys, OECD Pensions at a Glance (http://oe.cd/pag). China, Nigeria, Romania, Singapore and Thailand were not available and are approximations and are shaded in grey

Across all the countries people aged 50-54 made up around 10% of the working population, with Israel having a slightly lower percentage due to a younger population. For all countries, except for Singapore, China, Nigeria and Thailand (for which there are no data), the share of employment falls with age and shows a rapid decline after 65 years of age. The exceptions, Japan and South Korea, have the highest proportion of workers in both the 65-69 (6.4 and 4.4%) and 70-74 year old categories (4.1 and 2.5%). It can be noted that in the four countries without specific data, there are a large proportion of micro-enterprises with informal workforces characterised by lower education levels and often comprising many older people. Nigerian informal micro-enterprises account for more than 80% of total employment [6] and the older workforce, aged 50-69, make up 7.1%. In 2019, one-third (over 4 million people) of the Thai older population had remained in the workforce and 88% of older workers were informal [7]. The number of ageing workers in China (45 to 64) was over 290 million (38% of the working age population) in 2019 [8] with the majority of older workers being migrants from rural areas, with lower levels of education. Finally, in Singapore, 28.7% of older people are employed, constituting 7.2% of the workforce [9].

An official retirement age can only be regarded as indicative of the transitions that take place for people who are ageing. Retirement age may, for instance, vary between the private and government sectors and policy changes are occurring (generally increasing the ages of retirement) across the globe. Retirement age for both genders, however, hovers around 60-65 years across most of the fifteen countries. Nigeria, Thailand and China have the lowest retirement age (at around 60 years of age, and 55 for women in China). Israel has the highest retirement age for men but not women (67 versus 62 years) whereas, when both genders are considered, the US has the highest retirement age at 66 years.

### Australia

*Sabrina Pit, Winona Freihaut*

Australia is known as one of the countries to have been relatively successfully in combatting COVID-19 and has a high level of workforce participation among older people. The context is one where COVID-19 has given rise to increased online shopping and a movement to and creation of online businesses. In 2019, 75% of people aged 55-59 and 59% of people aged 60-64 were active in the labour force. However, COVID-19 has led to higher unemployment rates among older workers [10]. In 2021, a survey among Australian Human Resources experts found that 58% of older workers reported difficulty in finding employment or being retained at work during the pandemic [11]. Of interest is that smaller organisations were more likely to report that their organisation, to some or a greater extent, sought to retain their older workers in 2020 (42.5%) rather than medium (35.2%), large (23%) or very large organisations (23.7%). Additionally, lockdowns, international and internal state border closures have been occurring at different times across Australia. This has negatively impacted numerous Australian industries and businesses, leading to workforce participation challenges for older people.

It is well-known that a number of older people want to stay in paid employment or run a small business as opposed to retiring [12]. This highlights a need for an expansion of mature age employment and a transition to employment or entrepreneurship facilitation programs for older people.

‘Seniorpreneurship’ activities can be noted as rising in many countries, including Australia, Canada and the UK. People aged 50+ demonstrate the fastest growth in entrepreneurship in Australia [13] and 34% of all Australian small businesses are run by people aged over 50 years [13]. Seniorpreneurs are pointed to as providing many benefits to the workforce because of, for example,  having lower risk tolerance and a lower fear of failure, and a higher tendency to self-finance [13]. Maritz and colleagues posited that seniorpreneurs can potentially soften the negative impact of COVID-19 by leveraging their business experience, motivation and extensive networks.

In line with Maritz et al. [13] and Perenyi et al. [14], we argue further that policies and programs should be developed to promote seniorpreneurship. Governments can play a role here by raising awareness, providing training and education opportunities, business mentoring, funding opportunities, access to markets and networking. Encouraging a new field of self-employment can allow for continued workforce participation by older people. To this effect, Australia has previously funded initiatives to coach and support older people to start up new businesses in areas with higher proportions of older people.

Seniorpreneurs, however, have different needs from those employed by an organisation. Employers who encourage workers planning to retire to consider the seniorpreneurship alternative recognise this new model of working and its direct contribution to the maintenance of an age inclusive workforce - however some government or employer activities may inadvertently reach people with higher levels of income, education, privilege, skills and/or access to information - thereby further exacerbating inequities. Importantly, seniorpreneurship will be highlighted in the new International Standard on Age Inclusive Workforces being developed within the ISO.

### Austria

*Peter C. Huber*

At the beginning of 2020, 19% of the population of Austria was aged 65 years and older. The age structure will continue to shift in the direction of the 65+ group, exceeding 25% around 2030 [15], whilst the working-age population will shrink from 61% in 2020 to just over 50% [16]. These trends will have effects on areas including the labor market, health, housing and social services.

An Austrian survey [17] found that 42% of 208 respondent companies saw the effects of the pandemic on their company as ‘neutral’. Yet another 35% perceived the impact as ‘rather negative’. The survey also reported for the majority (63%) of older workers, that no changes had been made at work due to COVID-19, though 19% of companies offered workers the option to work from home. In any case, changes in working practices meant that 15% of workers had reduced personal contact with others and 3% were supported by younger co-workers.

Compared to a large proportion of older workers having no change at work due to the pandemic, it was those with a low or just above average monthly income, that were noted as facing with particular challenges. Many could only do their work to a limited extent or for a limited number of hours. With such challenges the potential consequences were noted in terms of changed rates of employment and loss of income, with the pandemic likely to further intensify social differences to the disadvantage of many older workers, unless more is done to enable them to retain and/or increase the extent of their employment.

Employee respondents were asked which strategies they were using to address challenges arising from the pandemic. In particular, virtual communication tools and the use of social media were mentioned. But, although these are now relatively commonplace, many older workers, particularly those on low incomes, may lack digital skills. A solution therefore may be to develop and offer free training to older workers, where necessary, to develop and accredit such skills. But the survey results pointed to the digital skills of some older workers sometimes being underestimated. Considering the amount of ‘home office’ work being done due to COVID-19, this is an important point to consider.

More widely in Austria, and in order to protect high risk groups, the government passed a law enabling people that were concerned to receive special paid leave. About 90,000 Austrians were registered for this - mainly those with medical problems and including older workers. They received letters affirming their higher-risk status which had to be confirmed by a doctor. It is plausible that, as a consequence, many of the older workers in the program will have been ‘sent’ into premature retirement. This could be regarded, depending on the length of the special paid leave, as a low-cost route (for companies) to facilitate early retirement for some of their workers. It remains to be seen whether this has a positive long-term impact on the employment or employability of older workers.

Except for a higher percentage of ‘home office’ possibilities compared to the period before the pandemic and the special paid leave option, no measures (aside from the government program to protect older workers and those with medical problems) were being taken to minimize the direct effects of COVID-19 on the older people’s employment. With some older workers retiring ‘early’, however, this will mean a loss of access (for employers) to a cohort of workers many of whom will have good experience and appropriate corporate knowledge. The long-term loss of income to those older people may, furthermore, not be in the best interests of both the Austrian society and the individuals concerned.

### Canada

*Rae Dulmage*

There are many Canadians aged 60 years and over who are semi-retired or still in full time employment and do both paid and unpaid work. The effects of COVID 19 on them are varied. It has delayed some people’s retirement and negatively affected the income levels of others. A case study illustrates the phenomenon:

Tom and Mark had both just retired as experts in their fields. In both cases their employer has brought them back to do work under contract since they cannot easily train or find new experts. Both had not anticipated the extra workload*.*

There were attempts by some organizations in Canada to downsize under COVID-19 conditions by retiring older and more expensive workers, but the Government put a stop to this practice. What was (and remains) a kind of devastation for the hospitality and retail sector during the pandemic was and continues to impact all age groups. Grocery prices increased and since interest rates are low (the prime lending rate has been at 0.6%) anybody with a low-risk investment portfolio is not receiving much benefit from savings or investments. While it is cheaper, borrowing is the last thing many older workers wants to do. On a more positive note there are different support measures in place that, while challenged because of the pandemic, are still working. Significant, however, is the problem for many older workers is when they have to care for a partner. The following case study demonstrates this.

An older Canadian needs a carer who is specifically trained in care procedures and what to monitor and watch out for. Currently one of the prime carers is off due to a work injury. There is only one other equally qualified carer employed by the service provider. She has stepped in to both train new staff and to work when others cannot. However, the care organization is unable to cope with the schedule or the qualification requirements of carers. The partner believes the system is fraying. The government has given the care recipient a $600.00 CAD one-time payment to offset some costs during COVID-19; and next year the Province Government is giving a write off of $2500 for home renovations related to the accessibility of the older person’s home*.*

More generally, older Canadians have also found it hard not to attend funerals of close friends or family that have died during the pandemic. This can affect people’s mental health and be something that disproportionately impacts older workers.

### China

*Qiang Hao*

The majority of the 293 million older workers (aged 45 to 64) work in low skilled jobs in sectors such as manufacturing, construction, transportation, warehousing, delivery, wholesale, retail, hotels and restaurants, and community services. About 41% of them have less than 9 years school education, but 76% have appropriate training for their jobs [8].

The impact of the COVID-19 pandemic had an immediate effect on ageing workers in restaurants. It was only one week from the most important national long holiday, the Spring Festival (Chinese New Year) in January 2020, when it was announced that the city of Wuhan would be locked down. Immediately, all restaurants in Beijing had almost no customers. The business losses were immediate due to increased stock and extra staff rostered for the expected peak of business over the Spring Festival period. Their costs were further increased because, by law, owners have to pay three times higher wages during public holidays. The outbreak of COVID-19, therefore, forced many owners to consider laying off staff or even closing their businesses. Older workers were the most vulnerable group among staff being laid off.

The Association of Restaurants in Beijing asked the municipal government of Beijing for help. The city’s government provided the following solutions:

1. Offering emergency funds. Low or no interest bank loans were offered to restaurants as emergency funds, provided they do not lay off staff.
2. Establishment of staff-sharing mechanisms. The city government also launched a project named ‘staff-sharing platform’ that has allowed restaurants to share their staff with supermarkets or grocery stores who were short of staff because of the sudden increase in on-line orders. The restaurant workers could work in the grocery stores temporarily but still keep their legal employment with the restaurants. The grocery store paid an extra bonus or additional hourly pay as an incentive to the workers concerned. Some restaurants even became suppliers of grocery stores by offering semi-cooked food for customers. This was a win-win-win situation for different stakeholders.
3. Promotion of eating-at-restaurants to return to business as usual through issuing cash vouchers to ‘eat-in’ customers. In July 2020, Beijing removed its restrictions on quarantine. To restore the economy of Beijing, the city government launched another programme to encourage eating in restaurants. It worked with some selected restaurants that strictly followed the rules of social distancing and the reinforcement of mask wearing, by issuing cash vouchers to ‘eat-in’ customers. The funds were intended to encourage the restaurants to return to ‘business as usual’.

In summary, local government intervention has played a key role in dealing with the COVID-19 emergency and business sustainability. Specific elements of their initiatives have helped support older workers.

### Germany

*Thomas Linner, Jürgen Deller, Anne Burmeister*

In the German labor market, the number of employees with reduced working hours in April 2020 reached 6.8 million, up from 133,000 in February due to COVID-19. In March and April, the number of jobs subject to social insurance contributions decreased by about 371,000, suggesting a reduction in employment, and in the number of part time jobs by 478,000. For June, an increase of 638,000 in the number of unemployed people was expected. The hospitality industry was hit hardest [18].

What influence did the COVID-19 pandemic have on older workers, who are amongst the highest risk groups? Westermeier [19] reported that, first, older workers are more satisfied with the government’s risk management practices than are younger workers. An important aim of the containment measures has been to protect workers through promoting reduced physical contact, virtual meetings and ‘home office’. And while stereotypes may suggest that older employees struggle with technology, research shows that they are more technologically savvy than was the case in the recent past [19]. Second, the unemployment rate has risen less strongly among older workers (aged 55 and over) than in younger age groups. The context is one where political decisions and company practices have created favorable conditions with flexible regulations so that people’s employment aspirations can be better realized at older ages. Third, the sharp decline in insecure employment suggests, according to Westermeier [20], that younger, older and low-skilled workers in particular have lost part of their income. Fourth, older employees work in jobs that require high mobility less frequently than their younger colleagues, meaning a higher proportion of older workers were able to continue working during the pandemic. Fifth, the reduction in working hours is only slightly greater in the 60+ cohort compared to younger groups - though there is no evidence that older people reduce their working hours to a particularly large extent in order to protect against health risks. And sixth, the extent of what is normally a higher health risk for older people due to the pandemic, differs according to the particulars of their work circumstances e.g., by virtue of the place of work, length of working hours, length of sick leave, or wages.

**Fig. 2:**
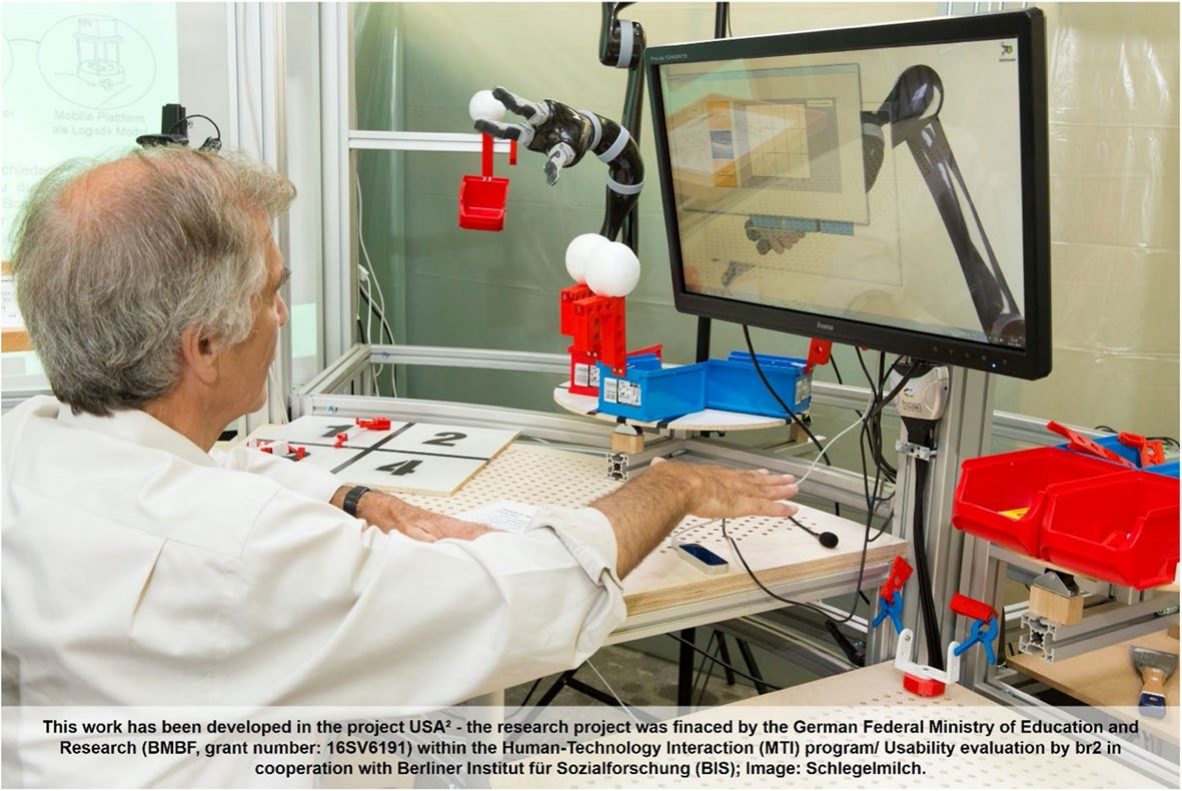
Age inclusive manufacturing system solutions: future workstations for older workers

Overall, the COVID-19 pandemic seems to have had less of an impact on older workers in the German labour market compared to younger, lower-wage employees. At the same time, interesting initiatives in workplace design that can help to support older workers are being promoted. One example is the multi-partner research project MAIA (Models and Methods for an Active Ageing Workforce: An International Academy) [21] that has developed some novel concepts and technologies for next generation age-inclusive manufacturing systems (see Fig. 2). This heralds the possibility that some older workers will be able to work to a later age and have a better opportunity to transfer their expertise to younger generations.

In order to tailor such workstations to the needs of the target group (older workers) these are being prototyped and tested in both laboratory and factory environments. They can assist with safeguards around COVID-19 on the shop floor level (taking account of requirements for hygiene, health monitoring, distance, intelligent work coordination, etc.).

MAIA built on previous work done in a German manufacturing industry context. Technology development tests have been conducted in the robotics lab at Technische Universität München (TUM) where the workstation was prototyped and a real-world assembly scenario with seven older people simulated. Open questions were addressed and behavioral analysis undertaken, as well as the exploration of different scenarios and functions.

In cooperation with the Berliner Institut für Sozialforschung GmbH (Berlin Institute for Social Research) and the development team at TUM, a further usability test was conducted. A key element of this was not only the evaluation of the individual functions of the workstation itself and the work procedures but also an evaluation of different control modes (for example semi-automatic, manual, gestures, voice, etc.). 21 older people took part in this usability study [22].

Notable is that the workstation can also assist, during the pandemic, through continuously monitoring the worker’s vital signs to detect potential health risks. And through scheduling work processes, the occupancy rate in specific work locations (not at home) can be reduced.

### Israel

*Peter Honeyman*

Israel is a young country. Birth rates are the highest in the OECD (3.2 per woman), with 27.5% of the population under 15, and only 10.5% aged over 65. Life expectancy is high at 82.6 [23]. Whilst the official retirement age for men is 67 and 62 for women, 22.4% of people over 65 are in employment. About a third of these state that this is due to the high cost of living relative to pensions and other support, their need to maintain their living standards, and to maintain interest [23]. When the COVID-19 pandemic hit, the Israeli economy was performing well, with Gross Domestic Product (GDP) growth being 3.4% in 2019, a record low unemployment level (3.8%), and relatively low public debt [23]. GDP per capita was above such countries as the UK and France. Israel was therefore in a relatively good financial place to respond to the pandemic. However, the country has major underlying challenges with high levels of poverty, large income gaps and wide disparities in productivity between the high-tech and older underperforming segments of industry. Within the OECD, in fact, Israel has the third highest proportion of poor workers, a disproportionate number of whom are women [24].

The government introduced partial wage support (at 70% of earnings) for those laid off because of the pandemic (i.e. a furlough scheme). However, for some industry sectors such as airlines and travel, tourism, and hospitality it is anticipated that there will long term effects,. The adjusted unemployment rate has risen to 4.9% by end of August and was projected to rise to 6.1% by the end of 2020 [23]. This excludes those employees on wage support.

There is no specific data on how many workers over 65 are receiving support or have been made unemployed. It is clear, however, that women have been more disadvantaged than men [25]. In addition, the government tried to ban workers over 67 going into any workplace during the pandemic. However, this proposed restriction was thrown out by the High Court in view of it being discriminatory [24].

At the beginning of the lockdown the general feeling was that Israel would adapt. People were used to work disruptions caused by irregular army call ups (that continue for people aged to 45) and the impact of occasional short wars.

Some workers in the employment system have benefited from an employer funded 7.5% tax free contribution towards continuing learning (Rifka [26]). In addition, some employers also fund sabbatical leave every 7 years. These measures value continuing human capital development and are of benefit to older workers. Some such older workers are employed in high tech, medical, administrative and knowledge industries and have been able to adjust to the pandemic by working from home. And in some specialist fields demand has increased.

In summary, Israel has a relatively low population aged over 65. About a quarter of these are working. Those with high human capital are the most resilient to shocks and disruptions. The government’s ability to support those on furlough protects the others. There has, however, been some discrimination against those on furlough in retirement age, and this has affected women more than men.

### Japan

*Hajime Yamada*

Japan is a ‘super aging’ country. In order words the rise in number of older people is particularly rapid. But there are many ‘living labs’ in Japan where older citizens actively participate in community-based platforms for open innovation and co-creation in collaboration with other stakeholders. An example is provided by the living lab. which was launched in 2017 in Kamakura City 50 km away from Tokyo. Older citizens organized the living lab to make working from home easier, regardless of age.

In the Kamakura Living lab many stakeholder organizations joined including the city authority, an academic institution and dozens of companies. Citizens, mostly older people, also joined with a view to resolving some of the everyday problems they encountered, such as those that resulted from the migration of young people to the cities. The research group focused on the living lab was led by Prof. Hiroko Akiyama, a gerontologist and founder of the Institute of Gerontology, University of Tokyo. The city authority participated with the aim of solving administrative issues and revitalizing the area. Companies also participated so that they could use it as an experimental forum to develop products and services that are more user-centric. Finally, university researchers, who wanted to study the effectiveness of the co-creation approaches that were adopted by various players, joined the living lab.

The context was one where young people, aged below 30, were increasingly leaving the area because of long commuting time to their workplaces in business districts of Yokohama and Tokyo. The living lab, following a series of brainstorming discussions, aimed to foster the creation of an environment where people could work in the Kamakura area but remotely from the city business districts. Two particular initiatives were implemented.

Firstly, practical measures were put in place for remote working through the setting up a shared office using an unoccupied shop in the area. A workable business model for this is still in development. Secondly, the need for functional ‘home office’ furniture for remote work was identified. Because Japanese houses are relatively small, workers need to be able to switch between work and personal life in a single room. A functional desk development project started from concept design, to trial production and usability testing by the Living Lab members. The new desk, named ONOFF, is compact enough so that the desk board can be easily folded up during non-working time. It is easy to use by any worker, regardless of age.

With the spread of COVID-19, remote working has been rapidly adopted in Japan. Many workers of all ages have been working from home. The compact desk for remote work developed at Kamakura Living Lab has captured a large market share and media attention.

Importantly, older people have contributed actively to the Kamakura Living Lab during the pandemic. There are two key success factors. Firstly, the design of the desk took into account the needs and opinions of living lab members. Secondly, the universal design concept was applied so that the desk is usable by not only older but also younger workers. Finally, the lessons learned were summarized from the perspectives of ageing workers for whom social participation is recognized as an important contributor to healthy ageing.

Given that employment is a form of social participation, older people who participate in the living lab can be considered to have undertaken project-based employment. It is, therefore, contributing to healthy ageing.

In summary, the compact desk helps to facilitate remote work for the older workers. Its benefits reflect their participation in its design and development. In addition, the desk has also been used by younger workers and so is helping to curb the local population decline that was a consequence of their leaving the area.

### Nigeria

*Bamidele Aloko, Fashola Akintunde, Mofoluwaso Nwamara*

COVID-19 has had a disproportionate impact on the ageing workforce in Nigeria. For the purpose of Nigeria, the ageing workforce is defined as persons aged 50 – 69. This takes account of the fact that retirement age in both the private and public sector is generally 60 years, though some categories of public servants retire at 65 or 70.

According to data published by the Nigeria Centre for Diseases Control (NCDC), older age groups account for a disproportionately higher percentage of reported COVID-19 cases and deaths. While the ageing workforce accounts for a mere 7.1% of national population, they account for 18.3% of COVID-19 cases recorded in the country and 54.1% of deaths. Retired workers (aged 70+) account for just 2.9% of COVID cases in the country, but 27.1% of deaths. With a case fatality rate of 10.6%, they are the most vulnerable. Table 3 shows statistics of cases and deaths per age band as at January 31, 2021.

**Table 3:** Statistics of cases and deaths per age band as at January 31, 2021

	Age Bands	No of Cases	No of Deaths	Case Fatality Rate	% Share of cases	% Share of Deaths	% Share of National Population^a^
**Retired**	70+	3163	335	10.6%	2.9%	27.1%	2.5%
**Ageing**	50-69	19,949	668	3.4%	18.3%	54.1%	7.1%
**Active**	20-49	72,944	215	0.3%	67.0%	27.1%	38.1%
**Dependents**	0-19	12,803	18	0.1%	11.8%	1.5%	52.3%

^a^ Nigerian Bureau of Statistics, 2021

Earlier during the pandemic, many of the country’s mostly informal 41.5 million micro enterprises (96% of all businesses in the country which account for more than 80% of total employment) had to either close or scale back their operations [6]. This necessitated the adoption of remote working. This would have affected many older workers, some of whom may not be tech savvy and therefore less able to use the technological solutions available.

Currently there are no data on government support specifically targeting the ageing workforce in Nigeria. Also, we have not been able to find any evidence of employers introducing measures specifically targeting this vulnerable group of employees. However, the government has issued advice and statistics highlighting the fact that older people are a vulnerable group and it has imposed lockdown measures, mandatory wearing of masks, closure of high-risk businesses and events, temperature scanning in public areas and the use of sanitizers and hand washing. Regular briefings by the Nigerian Centre for Disease Control are aired live on television.

Across both the public and private sector, offices have been closed in some parts of the country with only senior civil servants and staff on essential duties required to be present at work. However, the emphasis on seniority means that older, more vulnerable employees are more likely to be at work.

Other measures by the government include reducing the Value Added Tax (VAT) for small businesses, and along with NGOs and private sectors parties, rolling out measures where and when needed.

### Romania

*Adriana Ciacâru*

The pandemic has affected different sectors in many ways. From the restrictions imposed on the hospitality industry (where people had to find innovative solutions and take difficult decisions for their workers and businesses) to legal provisions which have impacted work life and the ability to work remotely. At the beginning of the pandemic, legal provisions made it mandatory for workers in the care system (including aged care facilities) to spend 14 days in isolation at the workplace. Many of these workers are older. This led to Federation Columna, the only Romanian trade union representative of the social assistance sector, taking several actions.

A preventive measure of two weeks isolation at work was put in place by the government [27] for people employed in social and child protection facilities. They were the only workers obliged to leave their families and homes for such a period, and subsequently were required to isolate for two weeks when returning to work. This has negatively impacted both their lives and their mental health.

The government tried to compensate for such strict measures by offering a risk incentive to these workers (Art. 7 from Law no. 82/2020). However, the disbursement for workers has awaited approval, this in turn, being dependent on reimbursement from European funds.

Another challenging aspect is a mandatory fortnightly COVID-19 testing process that care workers have been exposed to. The government has issued a new law that has increased mandatory testing frequency to weekly [28]. It is recognized that older people remain at increased risk of COVID-19 but the weekly testing impacts workers of all ages and, in particular, those who are older.

### Singapore

*Jason CH Yap.*

Singapore has one of the fastest ageing populations in Asia, with an estimated 15.2% of the population being aged 65 and above. Some 28.7% of older people are still employed, constituting 7.2% of the workforce [9].

Challenges for older people in Singapore have been no different from those that affect older people across the world. The country, fortunately, has been able to bring community transmission of COVID-19 down to the minimum. Initiatives in response to the first two waves were targeted towards international visitors and returning residents respectively; then being supplemented by a lockdown (called a ‘circuit breaker’) that was applied across the whole population. The third and largest wave of COVID infections was among migrant workers who generally had little contact with older people. This wave of infections was combated through focused containment measures [29].

Most measures for older workers have been aligned with the recommendations for vulnerable people more widely (i.e. pregnant women, older people, the immunocompromised, etc.). The Ministry of Health provided much guidance on ‘safe distancing’ (Singapore’s term for social or physical distancing), personal hygiene and other measures [30, 31]. The Ministry of Manpower, meanwhile, recommended that companies actively enable all employees to work from home where possible, especially those aged 60 and above and the immunocompromised. If the job roles of vulnerable employees could not be undertaken from home, employers were advised to redeploy the workers to other roles which could. If this was not possible, they did allow for these employees to work on company premises, but the company was then instructed to ensure that ‘safe management measures’ (for example physical and temporal spacing of persons, work areas and workstations) were well implemented. These were mandated for all workers irrespective of age, and included migrant workers [32].

In healthcare facilities, there was an even greater awareness of the risks involved in front-facing roles (i.e. dealing face to face with customers or others). Hence, besides implementing higher standards for infection control and measures for containment, some healthcare organizations discouraged staff over 50 years of age from volunteering for front line COVID-19 activities.

There were particular concerns about certain sectors, for example in the cleaning industry which employed significant numbers of older workers. Their risks were exacerbated by the increased cleaning activities because of the pandemic. One report pointed to the need for longer or more flexible working hours and larger areas to be used in order to clean more frequently as control measures were stepped up. There was great care however to ensure more than adequate personal protection, e.g. double gloving [33].

In summary, the main measures for the older workers are essentially the same as those applied to the general population, with extensive supporting policies and an infrastructure to ensure adequate execution. Case fatalities in Singapore are a mere 28 out of the initial 57,975 COVID-19 cases [34] and community transmission has been restricted [30, 31]. The best protection for older people in the workplace is the control of transmission in the community.

### Thailand

*Kamolpun Punpuing*

From the Elderly People’s Employment Survey (2019), 79.7% of older workers had levels of education lower than elementary; 58.7% were skilled agricultural and fishery workers; and 88.0% were informal workers. Thailand’s National Statistics Office (NSO) defines informal workers as those ‘who are not protected or have no social security from work’ [7]. After the age of 40, gradually more Thai people become informal workers [35].

During the lockdown in April 2020, older workers were among the first group asked to stop working and stay home due to the COVID-19 risk. Some employers asked such workers to transfer to other work sites, change to new work positions, or work online. Some were placed on paid or unpaid leave. Some business closures occurred. Consequently, many older workers adjusted their working lives by changing occupations (e.g. by helping in the family business), learning new skills, creating an online business, or training in electronic commerce [36].

Business shutdowns in the big cities led to the migration of many younger workers who moved back in with their parents in rural agriculture areas. The agriculture sector, however, suffered from socio-economic problems that arose due to drought, debt, a lack of resources and environmental challenges. COVID-19 exacerbated such problems and particularly impacted older people. Worsening the economic challenges was the fact that agricultural products cannot be easily sold in a timely manner because of temporary logistics and transportation restrictions. Some producers of these products could not adapt easily to sudden customer behavior changes and the need to find new marketing channels. At the same time, supplementary incomes derived from outside agricultural activities decreased due to layoffs, reduced working hours and less money being sent home from other family members, this leading to increased household debts. Coping strategies adopted by older people and/or their families were living off savings or assets, working more, reducing consumption, asking for help from relatives/ friends, and borrowing money from formal and informal systems [37].

Interestingly, during the lockdown, there were cases of intergenerational solidarity. Younger workers in the most affected services (including the tourism industry) explored new career opportunities. For example, they taught older workers about e-commerce or worked with older small business owners. COVID-19 and the influx of young people to rural areas was (and remains) an opportunity to build a new generation of agriculturalists with digital skills whilst also transferring the traditional agricultural knowledge from older farmers.

To counter the impact of COVID-19 pandemic, the Royal Thai Government has implemented economic measures to alleviate both the direct and indirect effects of the pandemic on the economy. There are measures that have focused on those workers outside the social security systems and on entrepreneurs [38] and include helping informal workers without social security benefits (including temporary employees and freelancers) and COVID-19 affected businesses. The measures have directly benefited older workers and business owners (See Appendix).

Other measures have been water and electricity fee reductions and an income support scheme for farmers. The Ministry of Labour also implemented measures such as encouraging part-time jobs, freelancing, and home-based work; providing reduced interest rates for home-based work loans, training courses on e-commerce; and paying unemployment and old age benefits under the Social Security Fund.

However, it is vulnerable older workers and small agricultural households who may have the greatest needs; who have not been able to access government benefits due to having no or low levels of education and digital skills; and have less supportive social networks. This highlights a widening wealth distribution gap in Thai society.

Future concerns for older Thai workers are about their ability to re-enter the workforce, how to manage digitalization of work and support their adjustment to new ways of working. Long-term strategies for ensuring worker security and benefits are needed. Importantly, future directions could usefully focus on the intergenerational approach, financial literacy, health literacy, digital skills development and further preparations for the ageing workforce.

### South Korea

*Tae Hwa Han*

Prior to the pandemic, older people in South Korea had been facing difficulties in maintaining jobs and livelihoods in the context of a rapid growth in the aging population and a weak social safety net. During the pandemic, South Korea’s older workers faced two major difficulties with the change to remote work as an alternative to unemployment. Firstly, it was not easy for many of them to access and use digital tools. Secondly, an increasing number struggled with depression as a result of prolonged isolation.

In March 2020, a government program funded jobs for older workers, where 83% (a total of some 534,000) of workers were laid off due to safety concerns caused by the virus. Two months later, in May, about 250,000 jobs were resumed, following the implementation of social distancing [39].

When the situation worsened and the number of COVID-19 cases increased rapidly, most businesses and institutions implemented remote work and flexible work arrangements. But with the new and unfamiliar non-face-to-face method of working being less accessible for many older workers, the South Korean government launched several online programs to promote the transitions. Fears have remain, however, because of many older people being unfamiliar with digital platforms and having poor understanding and utilization of digital technology. Such initiatives may, as a consequence, widen the gap in service access capabilities [39].

About 40% of the 684,000 people diagnosed with severe depression in Korea (announced by the Health Insurance Review and Assessment Service in 2018) were people aged 60 or over. Since the COVID-19 outbreak, not only have older people not been able to gather in public places such as parks (where they would often get together and chat with friends), but they are disproportionately facing mental health challenges that arise from their isolation. It is well-known that living alone, lack of exercise and lack of social contact can worsen symptoms of depression [39].

Some companies in Korea have created solutions to assist older people who live alone. SK Telecom’s Aria AI system continuously tracks more than 3200 service users, the majority of whom are older and live alone. And although there are some privacy concerns, local social workers are able to help monitor older people who may be vulnerable and be alerted if they are inactive for 24 h or other risks are identified such as when noted as conducting worrying online searches [39].

Local communities have also stepped up to support older people. Gimhae City (South Gyeongsang Province) conducted a `psychological quarantine` programme to counter the effects of isolation in September 2020 [40]. And due to the persistence of the virus, Gimhae City Council developed mental health care protocols by which they were able to offer personal psychological counseling. Mental health staff visited local areas twice a week to provide such counseling on a one-on-one basis based on depression and stress tests. This was for residents with difficulty visiting mental health counseling institutions. The ‘Social Farm’ organization, furthermore, provides services such as care, education, and employment to the socially disadvantaged including older agricultural workers [41]. These services have promoted mental as well as physical wellbeing through the production and distribution of agricultural products, and the provision of vocational training, horticulture and community activities. Families and older people with mental disabilities are complaining of ‘Corona-blue’ (COVID-19 related depression), as they lack access to different services and activities due to, amongst other things, distance from care facilities and the closure of some older people’s homes. Thus, as a countermeasure, the government currently operates 17 social farms for disabled people, 15 of which offer therapeutic programmes to overcome Corona-blue.

At the same time, the government is also supporting a ‘Farming Helper’ project to increase farming activities where most participants are older. `Farming Helper` supports (often older) farmers who have difficulty in farming due to accidents or illness, maintaining their livelihoods. The government stated it would actively support farming and continue to support social workers, the socially disadvantaged and their families to expand the farms and create a society where the socially disadvantaged are not excluded.

In conclusion, older workers in Korea are struggling when compared to younger workers due to the sudden need to adapt to changes in the working environment, and the depression caused by prolonged COVID-19 circumstances. The government is trying to address these problems with support from local governments and larger companies.

### Sweden

*Stefan Lundberg*

**Fig. 3:**
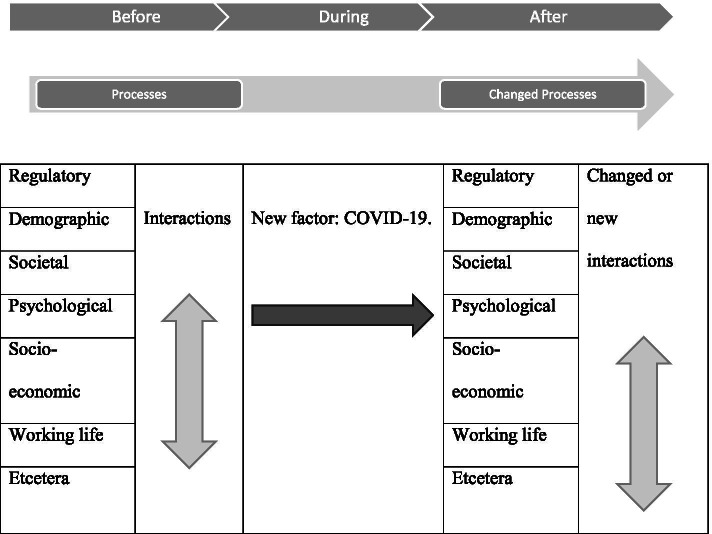
The Accident Theory Model

**Background:** A simple model based on a modification of accident theories can assist in addressing COVID-19 work-related issues (see Fig. 3). The COVID-19 ageing workforce model describes different process areas that are constantly evolving and interacting with each other. What happens to the older worker when a new unknown factor is introduced such as COVID-19? How does COVID-19 influence the various processes and their many complex interactions? Selected processes will be described at a high level, based on the Swedish situation with the older worker as the central focus.

**Regulation:** Most Swedish regulations concerning COVID-19 are issued as recommendations, not as compulsory laws. For example, ‘*keep your distance and take personal responsibility*’ [42]. Unlike many other countries, they are not linked to sanctions such as fines. Sweden did not initially go into lockdown like most other European countries, including its neighbors. It expects citizens to take personal responsibility for their behavior during the course of the pandemic. Of specific relevance to older people, the Swedish Public Health Agency decided in October 2020 to remove the special recommendations that people aged 70 years and older should generally avoid contacts with others, due to the poorer mental health that could be a consequence of their isolation. More generally, older people follow the same advice that applies to everyone.

**Psychological and societal issues: **The customs around personal space and proximity vary per country [43, 44]. In Sweden, cheek brushing/kissing and hugging is not common practice among older people. But it is conceivable that requirements around personal space might be increased in the future as a consequence of the pandemic.

The pandemic has also led to clear behavioural change [45]. It used to be uncommon for Swedes to order home food delivery, but now online orders for food and other goods have increased significantly [46]. Given that the risk of infection with more severe symptoms increases with age, more older people are ordering goods and services online. This has meant increased digital competence among older Swedes [47] which can also increase their employability [48]. Furthermore, home deliveries and online ordering has led to job creation e.g. as couriers.

**Working life:** Many people have increased working from home. However, the proportion of Swedes that avoided going to work peaked in early June 2020 at around 30%, with around 14% returning to their workplaces in October [49].

Teleworking places other demands on management, the work environment, and the boundary between work and private life. Overall, highly skilled workers (and ICT and knowledge intensive sectors) are better prepared for large scale telework. However, preparedness varies largely across EU countries [50]. The majority of European workers who started working from home, had not done so prior to the pandemic - although in Sweden, Finland and the Netherlands more than 30% of workers already worked regularly from home. Differences in ability to scale up telework could, however, lead to growing inequalities within organizations and across countries, impacting on different kinds of workers [50]. As a consequence, future employment contracts are likely to include updated rules on teleworking and working at home in order to avoid the risk of contracting or spreading COVID-19.

Some initiatives have been implemented to help Swedish workers of all ages. The Workers’ Education Association (Arbetarnas Bildningsförbund [51]) is Sweden’s leading study association. They ask: “*Are you laid off, or has your employment been affected by the corona pandemic in any other way?“*and they state *“Take the opportunity to acquire new knowledge that can facilitate your way back to work*”.

**The ongoing 'industrial revolution': **The first Industrial Revolution used water and steam power to mechanize production. The second used electric power to create mass production. The third used electronics and information technology to automate production. The fourth Industrial Revolution is characterized by the blending of technologies such as Internet of Things, robotics and connectivity, and the merging of the physical, digital, and biological domains. In this context it is likely that older workers will increasingly use health apps to monitor chronic conditions. The global COVID-19 pandemic may, in addition, trigger a new step in industrial development. The answer may not be that it will affect the older workforce positively or negatively, merely that it will be different.

### United Kingdom

*Malcolm Fisk*

Data from the United Kingdom (UK) Office for National Statistics (2020) shows the proportion of older people in work as steadily increasing from 4.8% in 2001 to 11.3% in 2020 (quarter two figures). This means that, in 2020, there were over 1,300,000 older people in full or part-time work. The growth in numbers has been encouraged by anti-age-discrimination legislation since 2006, and the removal of 65 years as the ‘official’ retirement age [52]. Being in work is, of course, a matter of choice for some and a necessity for other older people. Jobs, furthermore, can enhance or undermine people’s sense of health and well-being. Older people, as others, know and have experienced both.

The impact of COVID-19 on older workers will worsen with the ongoing pandemic and the ending of the UK government’s temporary furlough scheme (helping employers retain workers when closed or working below capacity). Two recent studies, both in collaboration with the survey company Ipsos MORI, help to set out the overall position.

The first study was undertaken by King’s College London in May 2020. It involved over 2200 UK adults aged 16-75 [53]. Analysis for those aged 55 to 75 found (with over 600 respondents) 2% having already ‘lost’ their job; and 25% who felt it was *more likely than not* (our emphasis) that they ‘will lose their job … as a result of the disruption from coronavirus’. Many of these will have benefited from the furlough scheme.

The second study (online, involving 1000 adults in England aged 55-74) was undertaken by the Centre for Ageing Better [54]. It pointed, drawing on other data, to 10 and 20% respectively of those aged 55-64 and 65-74 being ‘in insecure work’. A notable finding is the reported impact of COVID-19 on health, with nearly a quarter (23%) reporting this, including mental health, as having worsened.

For those in work it can be noted that (despite the legislation in place), a minority (9%) felt that their ‘employer treated me in a negative way because of my age’ and 12% felt they had been treated unequally. For those older people not in work almost 7 in 10 (68%) said they did ‘not feel confident that they will be employed in the future’ (this including 25%, of those furloughed). The study (also drawing on interviews with 19 older people) attested to older people feeling that ‘they were at an in-between stage – too young to retire but too old to be looking for work’. But over quarter (28%) of those aged 50-60 would ‘consider a career change’ and more were willing to change their working pattern.

A consequence of the pandemic has been the increase in home working. This can advantage many older people who may gain flexibility and be better able to manage (e.g. if they have mobility problems). The longer-term prospects for some of these, in the context of COVID-19, may be relatively good – depending on the sector in which they work. For those who lose their jobs, the safety net (aside from that which relates to personal investments, etc.) is restricted to welfare benefits and state pension entitlements. But it can be noted that there are job sectors with staff shortages that may be open to older workers who want to return and have the right skills or are willing to re-train.

The Open University Business Barometer [55], albeit before the pandemic, affirmed that ‘across the UK, 37% of organizations are looking to retrain older workers so that their skills are up to date’. Some sectors were noted with shortages (e.g. tourism) and clearly need to be reconsidered. But notable other areas of need (the need increased in some sectors due to the UK leaving the European Union) call for some skills and experience that many older workers can often bring. The need for health, social work and social care workers is particularly evident; so is the need for teachers, IT workers and skilled workers in the construction industry. It is these kinds of areas where the best opportunities for older workers may lie. And for some, e.g. nursing and teaching, there are incentive payments available.

In the short term, the position of many older workers in the UK is insecure. Their fears for the future of their jobs are fully justified. The context is one, however, where there is greater clarity emerging regarding future opportunities. Government support for older people seeking to work in (or return to) these sectors is warranted.

### United States

*Jennifer Schramm & Britta Berge*

Like the total US workforce, the 55+ worker population experienced high levels of unemployment due to the initial wave of COVID-19 and ensuing lockdowns. After peaking in April 2020 at 13.6% (12.1% for men ages 55+ and 15.5% for women ages 55+), the unemployment rate for people aged 55+ went down to 5.4% in October. However, despite the large drop in the unemployment rate and other signs of improvement in the 55+ labor force, experts on the aging workforce were concerned. They noted that, unlike in previous recessions when seniority and other factors helped keep unemployment rates lower for older workers than their younger counterparts, the current downturn seemed to be hitting older workers especially hard. In particular, older women, many of whom worked in the service sector and had jobs lost in the first wave of shutdowns, were experiencing very high unemployment rates.

Ghilarducci [56] analyzed data of the US Bureau of Labor Statistics data and noted that ‘unemployment rates for workers 55 and older have exceeded those of mid-career workers for the length of the pandemic - the first time since 1973 such an unemployment gap has persisted for six months or longer’.

While the pandemic-led downturn appeared to drive higher unemployment rates among older workers, the problems older jobseekers more commonly face during recessions persisted. Among them was the higher likelihood of long-term unemployment. In October, 31.7% of jobseekers ages 16 to 54 were long-term unemployed, up sharply from 18.2% in September. Meanwhile, 41% of jobseekers ages 55 and older were long-term unemployed in October 2020, having risen from 26.4% in September [57].

Along with long-term unemployment, older jobseekers routinely deal with age discrimination and the difficulty of recovering from the financial shock of unemployment. In 2015, several years after the official end of the Great Recession, AARP Public Policy Institute research found that many aged 45 to 70 were still trying to overcome challenges from their previous bouts of unemployment [58]. Another concern is a decline in the labor force participation rate among older workers and earlier than expected retirement due to the pandemic.

In April, the Coronavirus Aid, Relief, and Economic Security (CARES) Act, the largest emergency economic stimulus package in the history of the nation, was passed. This $2.2 trillion economic stimulus law was designed to fight the nationwide financial impact of COVID-19. The CARES Act extended unemployment insurance, offered small businesses money to pay their employees and sent many Americans $1200 cheques to stimulate the economy. However, many of these benefits had expired by the autumn of 2020, as lawmakers struggled to reach a consensus on further relief legislation. The early stimulus is the only coordinated US national effort related to jobs.

AARP, an association with nearly 38 million members, representing adults ages 50 and older, has partnered with the World Economic Forum (WEF) to encourage employers to embrace a multigenerational, inclusive workforce. Together they have launched an international learning collaborative which now includes over 40 companies. The Living, Learning, Earning, Longer (LLEL) initiative is based on the premise that, although governments can and should support the development of multigenerational, inclusive workforces, employers are best positioned to lead the charge. Employers who sign up to LLEL agree to adopt age-inclusive policies, enforce policies which prevent age discrimination, and support employees’ continued education and training.

Partnering organizations share resources and good practices, and collaborate on new research to help employers cultivate and sustain multigenerational workforces. For example, AARP has held a series of workshops including peer learning, and a webinar on *‘*Early Lessons & Promising Workforce Practices from COVID-19’ [59]. The learnings are being shared to combat the rising COVID-related long-term unemployment among older people and a digital learning platform is to be launched at the 2021 World Economic Forum Annual Meeting. It offers business cases and provides a guide for employers on how to support an age-diverse workforce, including relevant policies and practices.

Though the unemployment rate has declined from its peak early in the pandemic, it remains high for workers of all ages. Older jobseekers in the US are likely to face longer unemployment durations than their younger counterparts, and the age discrimination they routinely deal with could grow worse during this downturn. Although the federal CARES Act has provided temporary relief during the initial period of the pandemic, other large actors need to play a role to assist in ensuring long-term employability of the current workforce. The LLEL initiative is one example of an employer-focused solution including sharing good practices developed during COVID-19.

### Comparison

Table 4 shows some of the main COVID-19-related issues identified across the case studies and demonstrates the intersection between age with culture, socio-demographics, skills and industry sector.

**Table 4:** Main issues identified in relation to COVID-19 and the ageing workforce

Issue	Example (s)
**Cultural influences**	Israel reported that that Israelis are adaptable to sudden changes because of war conflicts. The emphasis on seniority in Nigeria means that older, more vulnerable employees are more likely to be at work whereas this may be the opposite in a Western country. Most Swedish regulations concerning COVID-19 are issued as recommendations, not as compulsory laws. Civic responsibility is easier to implement in a high developed social benefit state.
**Age population distributions and retirement vary.**	Japan being a super aging country whilst Nigeria and Israel being young countries. Overall countries have a relatively similar retirement age.
**More women disadvantaged**	Israel and the UK reported that women were more likely to be disadvantaged by the pandemic as many older women worked in the service sector, which remain one of the first industries to shut down in many countries.
**Lower income people more disadvantaged, often consisting of large proportions of older people**	Austria, Thailand and Germany reported that lower income people are facing particular challenges during the pandemic.
**Mental health impacts**	Mental health is large issue globally during the pandemic, reported e.g. by Romania, Sweden, South Koreas, UK and Canada. Case studies identified forced attendance at work for 14 days (Romania), increased depression due to prolonged isolation (South Korea), inability to attend import personal events such as funerals (Canada) and worsening mental health by a quarter of people participating in a survey (UK).
**Lack of digital skills**	Israel reported major underlying changes of large income gaps and disparities in productivity between high-tech segments and older, underperforming segments. Sweden also reported that highly skilled workers and ICT and knowledge driven sectors were most prepared to deal with change. This digital skills problem for workers in other sectors expands into their personal life; the ability to use online ordering and communicating online is important during the pandemic in order to maintain physical and mental health. While this overall remains an issue in Thailand, Sweden reports that the necessity of learning new digital skills has increased digital competence in the older population and thus may assist in future employability.
**Informal workforce**	Informal businesses and workforce play a large role in Thailand, Nigeria, China and South Korea. These workers are not socially protected in the same way as formal employment. Similarly, a UK study identified that a large proportion of older workers were in insecure work.
**Being laid off, once unemployed difficult to come back**	Being laid off from work is one of the most common issues reported due to COVID-19. China reported that older workers were the most vulnerable group among staff being laid off around spring time. Austria, Australia, UK, and the US reported that specifically for older people being unemployed makes it difficult to re-engage with work. Contrastingly, in Germany older workers were not particularly vulnerable to being laid off.
**Certain industry hit hardest**	China, Canada, Singapore and Israel reported that the services industry, including hospitality, cleaning and retail industries were highly impacted. It is anticipated that there will be long-term effects for these industries, as well as the travel and tourism industries.

Table 5 classifies the solutions applied in the case studies. The two major solutions focussed on economic measures and working from home. Other common solutions were cross industry collaboration to meet workforce shortages, promotion of online businesses, education and training, and intergenerational collaboration and product innovation.

**Table 5:** Solutions identified to improve the ageing workforce during COVID-19

Solutions	Examples
**Working from home**	• All Countries
**Laws and regulations mainly tied to economic measures**	• Paid leave for vulnerable workers (Austria) • Aid for informal workers, entrepeneurs, water and electricity fee reductions, farmer income support scheme (Thailand) • Aid for affected businesses (Thailand, US, Australia) • Program funding jobs for older workers (South Korea) • Partial wage support for unemployed/furlough scheme (Israel, UK) • Unemployment insurance extended, stimulus cheques (US) • Safety net for welfare benefits and state pension entitlements (UK) • Risk incentives for workers under strict conditions (Romania) • Payments to care recipients and for older person’s home accessibility (Canada) • Social farms for the disabled and psychological quarantine to counter isolation effects (South Korea) • Low interest loans when not laying off staff (China) • Consumer demand stimulation through cash vouchers for eat-in customers (China, Australia)
**Cross industry collaboration to meet workforce shortages**	• Staff sharing mechanisms across retail and hospitality led to innovation as some restaurants would provide cooked meals to grocery stores (China) • Job sectors with staff shortages that may be open to older workers who want to return and have the right skills or are willing to re-train such as health, social care, teachers and IT workers (UK) • The South Korean government launched several online programs to promote workers’ transitions to digital and non-face-to-face industries.
**Promotion of online business**	• Development of policies and programs to promote seniorpreneurship. COVID-19 has further emphasized the need for the government to create, support and grow locally owned businesses with a specific lens on seniorpreneurship. (Australia) • Older workers have been creating an online business (Thailand)
**Education and training**	• Governments can play a role in promoting seniorpreneurship by raising awareness, providing training and education opportunities, business mentoring, funding opportunities, access to markets and networking (Australia). • Free IT training (Austria) • Some workers in the employment system have benefited from an employer funded 7.5% tax free contribution towards continuing learning (Israel) • Older workers have received training in electronic commerce during lockdown (Thailand) • In South-Korea, the ‘Social Farm’ provides services such as care, education, and employment to the socially disadvantaged such as older workers in agriculture. • A series of workshops including peer learning, and webinars on ‘Early Lessons & Promising Workforce Practices from COVID-19’ [59]. The learnings will be shared to combat the rising COVID-related long-term unemployment among older people. (US)
**Intergenerational collaboration and product innovation**	• Multi-partner research projects: MAIA (Models and Methods for an Active Ageing Workforce: An International Academy) has developed some novel concepts and technologies for next generation age-inclusive manufacturing systems (Germany). • Many Living Labs exists throughout Japan in which older citizens actively participate in a community-based platform for open innovation and co-creation in collaboration with multiple-stakeholders. • Local social workers are able to help monitor vulnerable older people through using new products based on artificial Intelligence that continuously track older clients who live alone (South Korea). • Younger workers teach older workers digital skills, whilst older workers train younger people in agribusiness (Thailand). • AARP representing adults ages 50 and older, has partnered with the World Economic Forum (WEF) to encourage employers to embrace a multigenerational, inclusive workforce and launched a clearning collaborative: The Living, Learning, Earning, Longer (LLEL) initiative (US).

## Discussion

This paper has demonstrated some of the issues experienced by older workers as a consequence of the COVID-19 pandemic. Potential remedies to the disadvantages that have arisen are offered on individual, organizational, national and, potentially, international levels. While many countries pointed out that older people faced particular challenges, for example being more vulnerable to the virus, most measures for older workers simply aligned with the measures for vulnerable people in general.

Specific examples of solutions provided have included:

- Age inclusive manufacturing system solutions to provide future workstations for older workers;
- Living labs to solve local employment issues with older people as co-creators;
- Development of new business models (e.g. sharing of workers across different sectors);
- Encouraging senior entrepreneurship and business stimulation through policy, programs and funding opportunities;
- Provision of increased digital training and upskilling;
- Intergenerational skills transfer between urban young people (digital skills) and farmers (traditional farming methods);
- International learning collaboratives of organizations and companies;
- Local COVID-19 mental health programs and remote monitoring of older people;
- Job redesign and task shifting to reduce exposure to COVID-19 risk;
- Flexible working environments, including remote-work and flexible hours;
- Retraining of older workers to industries with workforce shortages such as health and aged care; and
- Reducing taxes, e.g. good and services tax for small businesses, and provision of tax incentives or funding schemes.

Several of the solutions provided relate to the circumstances that pertain in the particular countries and for which case studies have been offered. These case studies have demonstrated that the type and location of workplace, occupation and industry sector is key to variations in inequity and ageism. For example, in Romania two weeks isolation at work put in place for social and child protection facility workers and mandated workers to leave their families and homes behind during COVID-19 but it adversely impacted workers family life and mental health. In Thailand small agricultural households, commonly older people, have not been able to access many of the government benefits due to having lower levels of education and digital skills; and less supportive social networks.

The case studies have signaled themes or approaches that some of the countries have in common. Out of these, perhaps, three main concerns are evident.

These relate first to the fact that the COVID-19 pandemic is far from over. Older people remain the most vulnerable to infection, and, across all fifteen countries, have disproportionately been impacted by job losses and the restrictions that have been imposed. The second main concern relates to the likely increase in inequalities (partly fuelled by ageism) that is likely to result when the position of older people, and indeed older workers, is balanced against governmental or organizational resources and reduced revenue due to lockdowns and economic uncertainty. This is of particular concern in countries where governmental systems do not allow people to retire because there is a lack of benefits to take care of them. The third main concern relates to the notable differences between developed and developing countries. These differences relate to two issues. Firstly, informal businesses play a large role as noted in Thailand, Nigeria, China and South Korea. In Nigeria, over 40 million micro-enterprises, mostly with informal workers, represent 96% of all businesses and accounting for more than 80% of total employment. This calls for different solutions than those which are best applied in more developed nations. Secondly, in 2021 and beyond, COVID-19 vaccinations are being rolled out across the developed world with greater speed than developing countries, meaning that the especial vulnerability of older people may last for an extended period..

### Strengths and limitations

A major strength of this study is the inclusion of 15 countries and the diverse background of contributors with knowledge of matters pertaining to the ageing workforce, many of whom are themselves older people, and drawing from multiple disciplines and sectors (including standards, strategic policy and practice, consumer rights, HR, health and social care and industry). This diversity enabled recognition of a diverse range of solutions to help retention and improve the employability of older workers during and after the pandemic. We caution, however, that the specific solutions provided assisted in the earlier period of the pandemic and not all will necessarily help in the long-term. Special benefits paid to workers during lockdowns are unlikely, for instance, to be retained. Innovative services and products will also be less likely to assist those older people who are most in need due to many having less access to digital technology and with poorer digital literacy levels. Other limitations included the paucity in Africa and the absence of case studies from South America. The lessons learnt may, therefore, not be generalisable. Policy changes in many countries are, furthermore, occurring rapidly, such as the lifting of the retirement age (with China being one of the most recent planning to do so, through time, as part of a 2021 national plan). This may impact the findings of this study.

### Policy implications

As noted in the Background, the findings of this paper will inform work of the International Organization for Standardization Technical Committee 314 Ageing Societies (ISO TC314) in its development and implementation of the International Standard on age inclusive workforces. The paper contributes especially to the Sustainable Development Goals: good health and wellbeing; decent work and economic growth; and reduced inequalities.

The case studies’ have offered solutions which fit within strategies that have proven to work in reducing ageism [5]. These include:

- the introduction of policies and laws to increase benefits to workers due to lockdown (most countries);
- educational activities such as coaching and the promotion of ‘seniorpreneurship’ (e,g, in Australia); and
- intergenerational contact interventions (e.g. in Thailand with the reciprocal sharing of digital and agricultural expertise between younger and older people).

We suggest that policy makers, business owners, researchers and international organisations use the presented case studies and invest in evidence-based strategies to combat ageism and help ensure that the skills and knowledge of older people are recognised within inclusive workplaces. The case studies may present opportunities for further testing and scaling up of certain strategies. Collaboration between countries would assist in building the evidence base and upscaling these. 

More broadly it can be noted that the WHO has called for a campaign to change the narrative on aging [5]. The debate about how we create inclusive ageing societies and ageing workforces is, therefore, of crucial importance. It can be affirmed that the best protection, against COVID-19, for older people in the workplace is the control of transmission in the community. And although it may be unrealistic to resolve the social fabric issues during a pandemic, a global sharing of this new knowledge will assist in addressing the issues faced by older workers. It will enhance our ability to replicate some of the responses that have been indicated, and ultimately improve the quality of life of older people. Through the value of their retained skills or re-engagement in the world of work, knowledge sharing will support innovation, productivity and sustainability within organizations, ranging from farms to municipal and central governments.

## Conclusions

Several solutions to some of the problems faced by older workers in the context of COVID-19 presented have been identified. These vary from funding support schemes to encouraging business continuity, promoting innovative product and service developments, supporting community action and the development of new business models – for operation at local, national or international levels. The solutions can be seen as fitting within broader strategies that endeavour to reduce ageism and, in so doing, are supportive of ageing workers. They include policy and legal change, educational activities, and interventions to promote intergenerational contact. Global sharing of this knowledge will aid further understanding of the issues that are faced both by employing organizations and older people; and will facilitate the replication of solutions. Such action will thus help to reduce inequity, improve business continuity and the quality of life of older workers.

## Data Availability

Not applicable.

## Appendix

Government Implemented Measures for Thai Workers.

**Table:**

*Measures and remedies for salaried employees, fixed-term employees and self-employed workers that are not in the Social Security system, who are affected by the COVID-19 epidemic* • Income compensation to alleviate the burden for employees on permanent and fixed term contracts as well as self-employed workers who are not in the Social Security system or other people affected by the COVID-19. • Credit loan for expenditure programs for self-employed workers affected by the COVID-19 Virus (Emergency Loan). • Credit loan facility for expenditures for salaried employees affected by the COVID-19 virus (Special Credit Loan). • Low-interest loan facility for the Department of Social Development and Welfare to help low-income individuals who are directly and indirectly affected by the COVID-19 outbreak. • Knowledge enhancement measures; training to enhance professional skills or knowledge by interests, including the creation of a Corporate Social Responsibility (CSR) program. • Measures to postpone personal income tax payments • Measures to lift the deduction threshold for health insurance premiums • Personal income • Tax exemption as compensation for personnel at risk of occupational health and safety	
*Measures and Remedies for Entrepreneurs* • Introduction of a loan program to assist small entrepreneurs • Postponement of corporate income tax payments • Postponement of submissions and payments of tax returns • Tax filing extensions for industrial entrepreneurs who work in the oil industry • Tax filing and payment extensions for entertainment establishments • Exemption of import duty on items used for treatment, diagnosis or prevention of COVID-19 • Exemption from taxes and fees to support debt restructuring	
